# NEK1 Promotes Ovarian Cancer Progression via p53 Suppression While Enhancing Sensitivity to Genotoxic Therapy

**DOI:** 10.3390/cimb48050486

**Published:** 2026-05-07

**Authors:** Huiyang Song, Xia Wang, Aiqing Yang, Xuejiao Ren, Xiaoqi Zhou, Yifei Qiu, Yating Cai, Chengming Gao, Gangqiao Zhou, Pengbo Cao

**Affiliations:** 1 Academy of Military Medical Sciences, Beijing 100850, China; song_hickory@163.com (H.S.); 18589558796@163.com (X.W.); yan_gaiqing_ok@126.com (A.Y.); 3180101060@zju.edu.cn (Y.Q.); gchengming1988@163.com (C.G.); 2 State Key Laboratory of Medical Proteomics, National Center for Protein Sciences at Beijing, Beijing 100850, China; 3 Collaborative Innovation Center for Personalized Cancer Medicine, Center for Global Health, School of Public Health, Nanjing Medical University, Nanjing 211166, China; renxjy@163.com (X.R.); yting_cai@163.com (Y.C.); 4 College of Life Science, Hebei University, Baoding 071002, China; 15164542322@139.com

**Keywords:** ovarian cancer, NEK1, p53 signaling pathway, cell cycle, apoptosis, genotoxic treatment

## Abstract

Ovarian cancer (OV) is a highly metastatic and recurrent malignancy with limited therapeutic options. NIMA-related kinase 1 (NEK1), a serine/threonine kinase implicated in cell cycle regulation and DNA damage response, has been associated with tumorigenesis in various cancers, yet its specific role in OV pathogenesis remains elusive. This study systematically investigates the oncogenic function and underlying mechanisms of NEK1 in ovarian cancer. Our findings demonstrate that NEK1 promotes tumor progression both in vitro and in vivo. Mechanistically, bioinformatic and biochemical analyses reveal that NEK1 suppresses p53 signaling activity, resulting in downregulation of downstream targets p21 and PUMA, consequently attenuating cell cycle arrest and apoptosis. Importantly, NEK1-driven oncogenicity is dependent on the presence of p53 protein. Clinically, elevated NEK1 expression significantly correlates with poorer prognosis across multiple independent OV cohorts. Paradoxically, high NEK1 expression enhances radiosensitivity by impairing p53-mediated DNA damage repair. Collectively, these findings establish NEK1 as a promising prognostic biomarker and therapeutic target, with potential utility in guiding genotoxic therapy strategies for ovarian cancer patients.

## 1. Introduction

Ovarian cancer (OV) is a highly heterogeneous and recurrent malignant tumor, ranking as the eighth most common cancer among women [1]. Epithelial OV (EOV), accounting for approximately 90% of OV patients, represents the predominant histological subtype [1,2]. The pathogenesis of OV is driven by multiple factors, including genetic predisposition, age and hormone levels. For instance, women carrying *BRCA1* mutations exhibit an OV risk of about 40–65% [3]. Due to the unique anatomical position and structural features of the ovary, early-stage OV often presents with subtle symptoms, and highly sensitive screening methods are currently lacking. As a result, approximately 70% of patients are diagnosed only after the disease has progressed to extensive peritoneal dissemination or distant metastasis [4,5]. This delayed diagnosis contributes to a generally poor overall prognosis and poses a significant challenge to clinical treatment. Currently, the main treatment methods for OV include surgical resection and chemotherapy [4]. Although certain advancements have been achieved in these therapeutic approaches, high recurrence rates and unfavorable outcomes remain unresolved issues [6,7]. Consequently, elucidating previously unrecognized molecular drivers and tumorigenic mechanisms in OV represents a critical research priority, with significant potential to inform innovative therapeutic interventions for this aggressive malignancy.

NIMA-related kinase 1 (NEK1) belongs to the NEK family of serine/threonine protein kinases, which are critically involved in regulating the cell cycle and responding to DNA damage [8,9]. A previous study has shown that NEK1 could activate the CHK1 checkpoint and facilitate DNA repair through sustaining the phosphorylation of ATR [8]. Additionally, NEK1 also interacts with Ku80 to support non-homologous end joining, contributing to the maintenance of genomic stability [9]. Recent studies have associated NEK1 with tumorigenesis in multiple cancers, notably myeloma, glioblastoma, and carcinomas affecting the prostate, liver, stomach, colorectum, and esophagus [10,11,12,13,14,15]. NEK1 inhibitors significantly suppress the progression of glioblastoma [8]. Moreover, Knockdown of NEK1 in cervical cancer (CCA) and colorectal cancer (CRC) cells significantly enhances their sensitivity to radiotherapy [10]. Mechanistically, NEK1, when phosphorylated by TLK1, promotes prostate cancer progression and drug resistance [16,17]. Collectively, these findings suggest that targeting NEK1 may provide a promising therapeutic strategy for the treatment of various cancers and other related diseases. However, the biological functions and clinical significance of NEK1 in OV remain largely unknown.

In this study, we found that elevated NEK1 expression serves as a risk predictor in OV, as it significantly correlates with advanced clinical stages and poor outcomes. Functionally, our findings demonstrate NEK1 promotes OV tumorigenesis through suppressing p53 expression and signaling, which leads to downregulation of its downstream target genes, including p21 and PUMA. Notably, OV cells with high NEK1 expression show increased sensitivity to ionizing radiation (IR). Taken together, these results highlight NEK1 as a potential prognostic biomarker and therapeutic target for OV, providing a novel rationale for the development of NEK1-targeted strategies combined with radiotherapy in the clinical management of this disease.

## 2. Materials and Methods

### 2.1. Cell Lines

OVCAR-3 and ES-2 human ovarian carcinoma cell lines, together with HEK293T embryonic kidney cells and HCT116 colorectal cancer cells, were procured from the China Center for Type Culture Collection (CCTCC, Wuhan, China). Standard culture conditions comprised DMEM-based medium fortified with 10% fetal bovine serum and a penicillin-streptomycin cocktail (100 U/mL and 100 μg/mL, respectively), with incubation at 37 °C in a humidified 5% CO_2_ atmosphere.

### 2.2. NEK1 Knockdown and Overexpression Assays

The lentiviral small hairpin RNA (shRNA) constructs coding a scramble sequence and 2 independent sequences targeting human *NEK1* (*NM_001199397.3*) were obtained from Hanbio Co., Ltd. (Beijing, China; Supplementary Table S1). The pLVshRNA-EGFP(2A)-Puro lentiviral backbone (Inovogen, Chongqing, China) served as the cloning vector for shRNA insertions, with successful integration verified through Sanger sequencing. As a negative control, a scrambled shRNA lacking sequence homology to any annotated mammalian transcript was employed. Lentiviral particles were generated in HEK293T cells via triple-plasmid transfection (transfer vector:packaging plasmids = 3:2:1) using Lipofectamine 2000 (Invitrogen, Carlsbad, CA, USA). Following 8–12 h of viral exposure, target cells underwent puromycin selection (2 μg/mL) for 14 days. Knockdown efficiency was assessed by qRT-PCR and Western blot. For overexpression, full-length NEK1 cDNA was inserted into pLV-neo, sequence-confirmed, and packaged using the aforementioned protocol. Stable cell lines were established and validated as described above. For p53-dependency experiments, OVCAR-3 and ES-2 cells were transiently co-transfected with si*NEK1* (100 nM) and either si*TP53* (100 nM) or non-targeting control siRNA (siCtrl, 100 nM) by using the riboFECT CP Transfection Kit (RiboBio, Guangzhou, China). Knockdown efficiency was validated 48 h post-transfection by qRT-PCR assays.

### 2.3. Cell Growth, Plate Colony Formation, Migration and Invasion Assays

For cell viability, OVCAR-3 and ES-2 cells were plated at 2 × 10^3^ cells/well in 96-well plates. Following 6–8 h adherence, CCK-8 solution (ZP328, Zhuangmeng Biotech, Beijing, China) was added and incubated (37 °C, 1 h). Absorbance at 450 nm was read on a Tecan Spark microplate reader. Measurements were taken daily for 4 days. For colony formation, cells (1 × 10^3^/well) were seeded in 6-well plates and maintained for 14 days. Colonies were fixed in 4% paraformaldehyde, stained with 0.5% crystal violet (Solarbio, Beijing, China), and enumerated under an Olympus IX71 microscope. For migration, 8 × 10^4^ cells in DMEM/20% FBS were placed in the upper chamber of 24-well Transwell inserts (8 μm, BD Biosciences, 353097, San Jose, CA, USA); for invasion, 8 × 10^4^ cells were seeded onto Matrigel-coated inserts (BD Biosciences, 354480). After 24 h (migration) or 48 h (invasion), cells on the lower surface were fixed, stained with crystal violet, and counted in three random fields per well. All assays were performed in triplicate.

### 2.4. Functional Enrichment Analyses

For pathway analysis, differentially expressed genes (|log_2_FC| > 1, adjusted *p* < 0.05) identified from the TCGA-OV cohort were subjected to Gene Set Enrichment Analysis using GSEA software (v4.3.2) [18] against the MsigDB Hallmark gene set (v2023.1) [19]. Enrichment significance was determined based on normalized enrichment scores (NES) derived from 1000 permutations, with the false discovery rate (FDR) < 0.05 considered statistically significant.

### 2.5. Western Blotting Assays

Total cellular proteins were extracted using RIPA lysis buffer supplemented with protease and phosphatase inhibitors, and quantified by the BCA method. Equal amounts of protein (30 μg) were mixed with loading buffer containing 2-mercaptoethanol and denatured by boiling at 95 °C for 5 min. Samples were separated by 12% SDS-PAGE electrophoresis (100 V, 2 h), followed by electrotransfer to PVDF membranes (0.45 μm, 100 V, 90 min). The transferred PVDF membranes were blocked with 5% non-fat milk/TBST (20 mM Tris-HCl, 150 mM NaCl, 0.1% Tween-20, pH 7.6) at room temperature for 1 h. Primary antibody incubation: Membranes were incubated with corresponding primary antibodies (NEK1 1:1000, Santa Cruz sc-398813, Dallas, TX, USA; p53 1:1000, Proteintech 10442-1-AP, Rosemont, IL, USA; p21 1:1000, Abcam ab109520, Cambridge, UK; BAX 1:1000, Proteintech 50599-2-Ig; PUMA 1:1000, Proteintech 55120-1-AP; Abcam ab256469) overnight (12–16 h) at 4 °C with shaking. The following day, membranes were washed with TBST 3 times for 10 min each. Secondary antibody incubation: HRP-conjugated secondary antibodies (anti-rabbit IgG 1:3000, CWBIO CW0103M; anti-mouse IgG 1:3000, CWBIO CW0102M) were added and incubated at room temperature for 1–2 h. Membranes were washed with TBST 3 times for 10 min each. Detection and imaging: Enhanced chemiluminescence reagent (ECL, Millipore, Burlington, MA, USA) was used for detection, and images were captured using a chemiluminescence imaging system (Azure Biosystems, Dublin, CA, USA). Densitometric analysis was performed using ImageJ software (v1.53), with GAPDH (Proteintech 60004-1-Ig, 1:1000) serving as the internal reference for normalization [20,21].

### 2.6. RNA Isolation and Quantitative Real-Time Polymerase Chain Reaction (qRT-PCR) Assays

Total RNA was extracted using TRIzol reagent following the manufacturer’s instructions. cDNA was synthesized from 1 μg RNA using SuperScript III Reverse Transcriptase (Invitrogen, 18080051, Carlsbad, CA, USA). qPCR was carried out in triplicate on a QuantStudio 3 system (ThermoFisher, A28567, Waltham, MA, USA) with 10 μL reactions comprising KAPA SYBR FAST Master Mix (Roche, KK4601, 2×) and 0.2 μM gene-specific primers. Expression levels were normalized to GAPDH and calculated by the 2^−ΔΔCt^ method. Data represent mean ± SD of three biological replicates.

### 2.7. Cell Cycle Assays

Cell cycle distribution was assessed through flow cytometric analysis. Following trypsinization, cells were harvested and subjected to fixation in 70% (*v*/*v*) ethanol, subsequent to which propidium iodide (PI, 50 μg/mL) supplemented with RNase A (1 mg/mL) (Sigma-Aldrich, St. Louis, MO, USA) was applied for 30 min at 37 °C. Nuclear DNA content was subsequently quantified on a FACSCalibur instrument equipped with CellQuest software (version 6.0, BD Biosciences, USA).

### 2.8. Apoptosis Assays

Apoptosis was determined by dual-color flow cytometry following Annexin V-APC and propidium iodide labeling (Keygen, Nanjing, China). Briefly, cells were harvested and washed, then resuspended in binding buffer, followed by dual staining with Annexin V-APC and propidium iodide (PI). For each experimental group, a total of 8 × 10^5^ cells per sample were run on the FACSCalibur platform (BD Biosciences, USA), and fluorescence data were extracted using CellQuest acquisition software. Cells positive for Annexin V were gated and defined as the apoptotic population.

### 2.9. Nude Mice Assays

Female BALB/c nude mice (6 weeks old, Vital River Laboratories, Beijing, China) were maintained under specific-pathogen-free (SPF) conditions. Animal protocols were approved by the Ethics Committee of the National Center for Protein Sciences (Beijing). For xenograft studies, mice were randomized into experimental groups (*n* = 5) prior to tumor cell injection. For the subcutaneous xenograft model, OVCAR-3 cells with NEK1 overexpression and empty vector control (5 × 10^6^ cells resuspended in 200 μL PBS) were subcutaneously injected into the bilateral dorsal flanks of mice, respectively. Tumor volumes were monitored weekly using a vernier caliper and calculated based on two-dimensional measurements via the formula: Tumor volume (mm^3^) = (length [mm] × width [mm]^2^)/2. After 5 weeks of implantation, all mice were euthanized, and subcutaneous tumors were carefully dissected and harvested. Fresh tumor tissues were weighed immediately, followed by fixation in 4% paraformaldehyde, dehydration, and paraffin embedding. Subsequently, paraffin-embedded sections were subjected to hematoxylin and eosin (H&E) staining and immunohistochemical (IHC) analysis for subsequent phenotypic detection.

### 2.10. Immunohistochemistry (IHC) Staining Assays

Tumor tissues were fixed in 4% paraformaldehyde, paraffin-embedded, and cut into serial sections. Following xylene dewaxing and gradient ethanol rehydration, heat-mediated antigen retrieval was performed in EDTA buffer. Sections were blocked and incubated overnight at 4 °C with anti-Ki-67 primary antibody (Santa Cruz Biotechnology, sc-23900, 1:500, Dallas, TX, USA), followed by HRP-conjugated secondary antibody (2 h, RT). DAB chromogenic reaction and hematoxylin counterstaining were then applied. Imaging was performed using a microscope (Olympus, Tokyo, Japan). IHC staining results were assessed by two independent observers in a blinded semi-quantitative scoring system. Evaluation employed a dual-parameter scoring algorithm. Intensity of immunohistochemical signal was assessed using a 0–3 scale (0 = no staining, 1 = weak, 2 = moderate, 3 = strong). The fraction of positively stained cells was quantified across five intervals: 0 (<5%), 1 (5–25%), 2 (26–50%), 3 (51–75%), and 4 (>75%). Final staining indices (0–12) were computed by multiplying the respective intensity and cell fraction scores.

### 2.11. Bioinformatics and Survival Analysis

Gene expression profile datasets and ovarian cancer patient survival information were downloaded from The Cancer Genome Atlas (TCGA) and Gene Expression Omnibus (GEO), including datasets GSE63885 (Affymetrix HG-U133_Plus_2 platform), GSE26712 (Affymetrix HG-U133A platform) and GSE165808 (Illumina HiSeq 2500 (*Homo sapiens*)). The “survminer” package (https://github.com/kassambara/survminer (accessed on 1 May 2026)) was used to calculate the optimal grouping threshold for NEK1 mRNA expression in ovarian cancer patients to maximize the correlation between NEK1 expression and overall survival. Patients were stratified into high- and low-NEK1 expression groups accordingly. Kaplan–Meier survival curves were plotted, and differences between curves were evaluated using the log-rank test. Univariate Cox proportional hazards regression was performed to estimate hazard ratios (HR) and 95% confidence intervals (CI). Differential NEK1 expression between chemotherapy-response subgroups was assessed using the Mann–Whitney U test.

### 2.12. Statistical Analyses

ImageJ software (v1.53) was used for image acquisition and quantitative analysis, including Western blot densitometry, colony formation counting, and immunohistochemical scoring. FlowJo (v10.8.1) was employed for flow cytometric analysis of apoptosis and cell-cycle distribution. GraphPad Prism 10 was used for data visualization and statistical analysis. All quantitative data are presented as mean ± standard deviation (SD). Comparisons between two groups were analyzed using Student’s *t*-test (two-tailed, unpaired unless otherwise specified). Two-way ANOVA was used for repeated-measures data (e.g., CCK-8 proliferation assays). The Mann–Whitney U test was applied for non-parametric group comparisons. A *p*-value < 0.05 was considered statistically significant. Detailed methods for survival analyses are described in Section 2.11.

## 3. Results

### 3.1. High Expression of NEK1 Is Associated with Poor Clinical Outcomes

**Figure 1:**
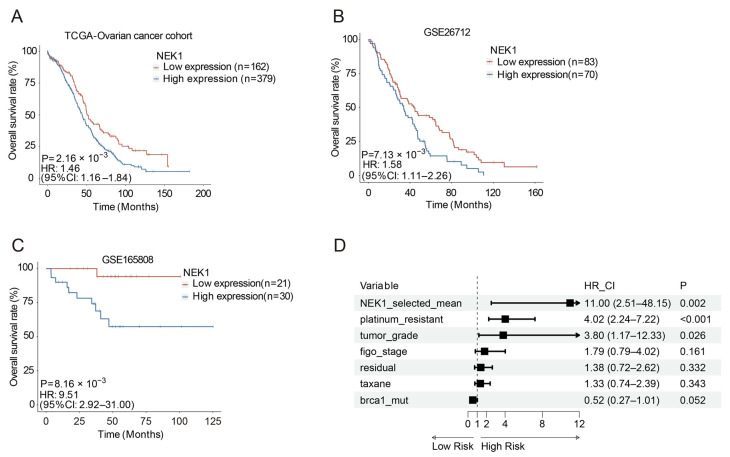
Elevated NEK1 expression predicts unfavorable clinical outcomes in ovarian cancer. (**A**–**C**) Kaplan–Meier survival curves for OV patients stratified according to NEK1 expression levels. The gene expression data and clinical information were obtained from the TCGA-OV (**A**), GSE26712 (**B**) and GSE165808 (**C**) datasets. HR with corresponding 95% CI were derived from Cox regression, with significance evaluated via log-rank testing. (**D**) Multivariate analysis of NEK1 mRNA expression and common clinical covariates in association with ovarian cancer survival in GSE63885. HRs and *p* values were calculated with a two-sided Cox proportional hazard model.

To investigate the relationship between NEK1 expression and prognosis, we analyzed an ovarian cancer cohort from TCGA and GEO Datasets (GSE26712 [22,23], GSE63885 [24,25] and GSE165808 [26]). The TCGA-OV cohort consisted of 609 ovarian carcinoma samples with RNA-sequencing data and comprehensive clinical annotations, including International Federation of Gynecology and Obstetrics (FIGO) stage, histological grade, and overall survival (OS). For external validation, we incorporated three independent microarray datasets: GSE26712 (*n* = 195, normal ovarian and ovarian cancers), GSE63885 (*n* = 101, ovarian cancer surgical sample), and GSE165808 (*n* = 51, high-grade serous ovarian carcinoma). Patients with increased NEK1 expression exhibited significantly compromised overall survival in TCGA (HR (95% CI) = 1.46 (1.16–1.84), *p* = 2.16 × 10^−3^), GSE26712 (HR (95% CI) = 1.58 (1.11–2.26), *p* = 7.13 × 10^−3^) and GSE165808 datasets (HR (95% CI) = 9.51 (2.92–31.00), *p* = 8.16 × 10^−3^) (Figure 1A–C). Furthermore, Multivariate Cox regression analysis of the GSE63885 dataset confirmed high *NEK1* expression as an independent predictor of unfavorable outcome, retaining statistical significance after adjustment for FIGO stage, tumor grade, platinum resistance, taxane treatment, residual disease and *BRCA1* mutation (HR (95% CI) = 11.00 (2.51–48.15), *p* = 0.002) (Figure 1D). Collectively, these findings indicate that NEK1 upregulation is strongly associated with adverse prognosis in ovarian cancer and suggest its potential as a molecular biomarker for prognostic stratification.

### 3.2. NEK1 Promotes Cell Growth, Migration, and Invasion of Ovarian Cancer Cells

**Figure 2:**
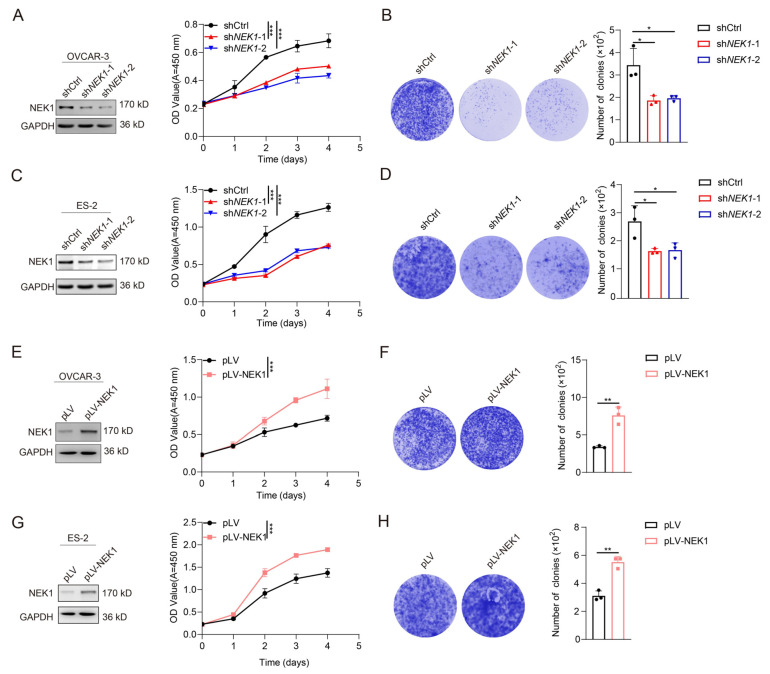
NEK1 overexpression promotes the growth of ovarian cancer cells in vitro. (**A**) OVCAR-3 cells were stably transduced with the shNEK1 plasmid or respective controls. Whole-cell lysates were immunoblotted with anti-NEK1 and anti-GAPDH antibodies. Effects of NEK1 knockdown on cell growth determined by CCK-8 assay in OVCAR-3 cells. (**B**) Effects of NEK1 knockdown on cell growth determined by colony formation assays in OVCAR-3 cells. (**C**) ES-2 cells were stably transduced with the shNEK1 plasmid or respective controls. After 48 h, whole-cell lysates were immunoblotted with anti-NEK1 and anti-GAPDH antibodies. Effects of NEK1 knockdown on cell growth determined by CCK-8 assay in ES-2 cells. (**D**) Effects of NEK1 knockdown on cell growth determined by colony formation assays in ES-2 cells. (**E**) OVCAR-3 cells were stably transduced with NEK1-overexpression plasmid or respective controls. After 48 h, whole-cell lysates were immunoblotted with anti-NEK1 and anti-GAPDH antibodies. Effects of NEK1 overexpression on cell growth determined by CCK-8 assay in OVCAR-3 cells. (**F**) Effects of NEK1 overexpression on cell growth determined by colony formation assays in OVCAR-3 cells. (**G**) ES-2 cells were stably transduced with NEK1-overexpression plasmid, or respective controls. After 48 h, whole-cell lysates were immunoblotted with anti-NEK1 and anti-GAPDH antibodies. Effects of NEK1 overexpression on cell growth determined by CCK-8 assay in ES-2 cells. (**H**) Effects of NEK1 overexpression on cell growth determined by colony formation assays in ES-2 cells. Data are shown as mean ± standard deviation (SD) of three independent experiments, two-tailed Student’s *t* test. * *p* < 0.05 vs. control, ** *p* < 0.01 vs. control, *** *p* < 0.001 vs. control.

Given that NEK1, a serine/threonine kinase, has emerged as a potential tumor promoter across various malignancies [1], we next sought to systematically investigate its functional relevance in OV development and progression. The oncogenic potential of NEK1 in OV cells was assessed using CCK-8 viability and clonogenic assays. Knockdown of NEK1 using short hairpin RNA (shRNA) significantly suppressed cell growth as demonstrated by the CCK-8 assays in OV cell lines OVCAR-3 and ES-2 (Figure 2A,B). Consistently, colony formation assays revealed that knockdown of NEK1 significantly reduced the clonogenic capacity of OVCAR-3 and ES-2 cells (Figure 2C,D). Conversely, ectopic overexpression of NEK1 significantly promoted cell growth and colony formation ability (Figure 2E–H).

Considering the importance of migratory capacity in OV metastasis [2,5], we next examined the effects of NEK1 knockdown/overexpression on ovarian cancer cell migration and invasion. Transwell assays were performed to assess whether the expression levels of NEK1 significantly affect these cellular processes. The results showed that NEK1 knockdown significantly inhibited the migration and invasion capabilities of OVCAR-3 (Supplementary Figure S1A) and ES-2 cells (Supplementary Figure S1B). Conversely, ectopic overexpression of NEK1 markedly promoted the migration and invasion capabilities of these OV cells (Supplementary Figure S1C,D). Collectively, these findings suggest that NEK1 enhances the malignant properties of ovarian cancer cells by promoting cell proliferation, colony formation, migration, and invasion.

### 3.3. NEK1 Promotes the Tumor Growth of OV Cells

**Figure 3:**
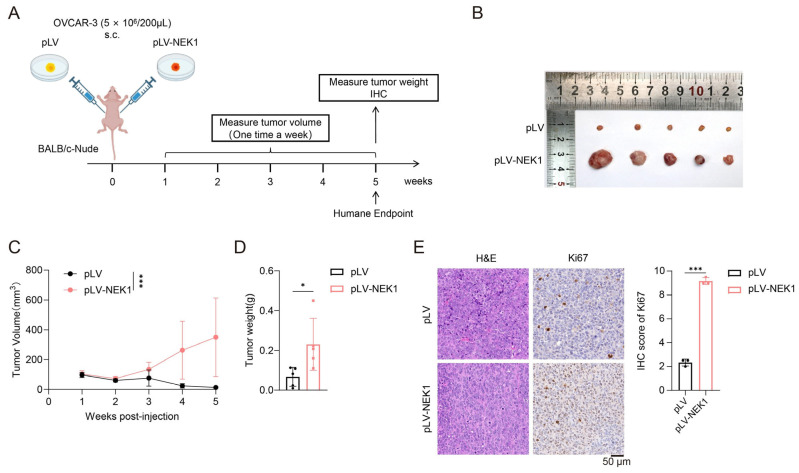
NEK1 accelerates tumor growth and aggravates the malignant progression of ovarian cancer cells in vivo. (**A**) Schematic illustration of subcutaneous xenograft tumor formation in nude mice. (**B**) Representative endpoint tumors from mice injected with NEK1-overexpressing (pLV-NEK1) or empty-vector (pLV) OVCAR-3 cells (*n* = 5 per group). (**C**) Tumor volume was monitored every week with digital calipers [(length × width^2^)/2] from week 1 until euthanasia (week 5). NEK1 overexpression (pLV-NEK1) vs. pLV: *** *p* < 0.001 from day 28 onward. (**D**) At endpoint (day 35, week 5), tumors with NEK1-overexpressing (pLV-NEK1) or empty-vector (pLV) OVCAR-3 cells were excised and weighed immediately. Data are mean ± SD, *n* = 5. Statistical significance was determined by an unpaired two-tailed Student’s *t* test, * *p* < 0.05. (**E**) Formalin-fixed, paraffin-embedded tumor Sections (4 µm) were stained with H&E or subjected to IHC using anti-Ki67. Positive signals were visualized with DAB and counterstained with hematoxylin. Representative images show elevated Ki67 and NEK1 expression in pLV-NEK1 tumors. Scale bars, 50 µm. *** *p* < 0.001.

To further investigate the oncogenic role of NEK1 in OV progression in vivo, we established a subcutaneous tumor-bearing model in BALB/c nude mice. First, we established stable cell lines with NEK1 knockdown or overexpression in OVCAR-3 and ES-2 cells, following the methods described in Section 2.2. Subsequently, as detailed in Section 2.9, OVCAR-3 cells stably overexpressing NEK1 or empty vector control cells were subcutaneously inoculated into BALB/c nude mice to establish a subcutaneous xenograft tumor model. OVCAR-3 cells stably overexpressing NEK1 or carrying an empty vector were injected subcutaneously into the dorsal flanks of nude mice, with subsequent continuous tumor monitoring. (Figure 3A). Consistent with our in vitro findings, compared with the control cells, NEK1-overexpressed OVCAR-3 cells showed significantly accelerated tumor growth, as evidenced by weekly measurements of tumor volumes (Figure 3B,C). At the end of the five-week observation period, the endpoint tumor weight in the NEK1-overexpressed group was significantly higher than that in the control group, confirming the promotion of tumor growth by NEK1 (Figure 3D). Immunohistochemical (IHC) staining analyses showed that the Ki67 labeling index (a well-recognized marker of cell proliferation) increased from 2% in the control group to 9% in the NEK1-overexpressed group (Figure 3E), further verifying that NEK1 enhances OV cell growth in vivo. Collectively, these in vivo data strongly confirm the oncogenic role of NEK1 in promoting OV progression.

### 3.4. NEK1 Reduces the Activity of p53 Signaling Pathway

**Figure 4:**
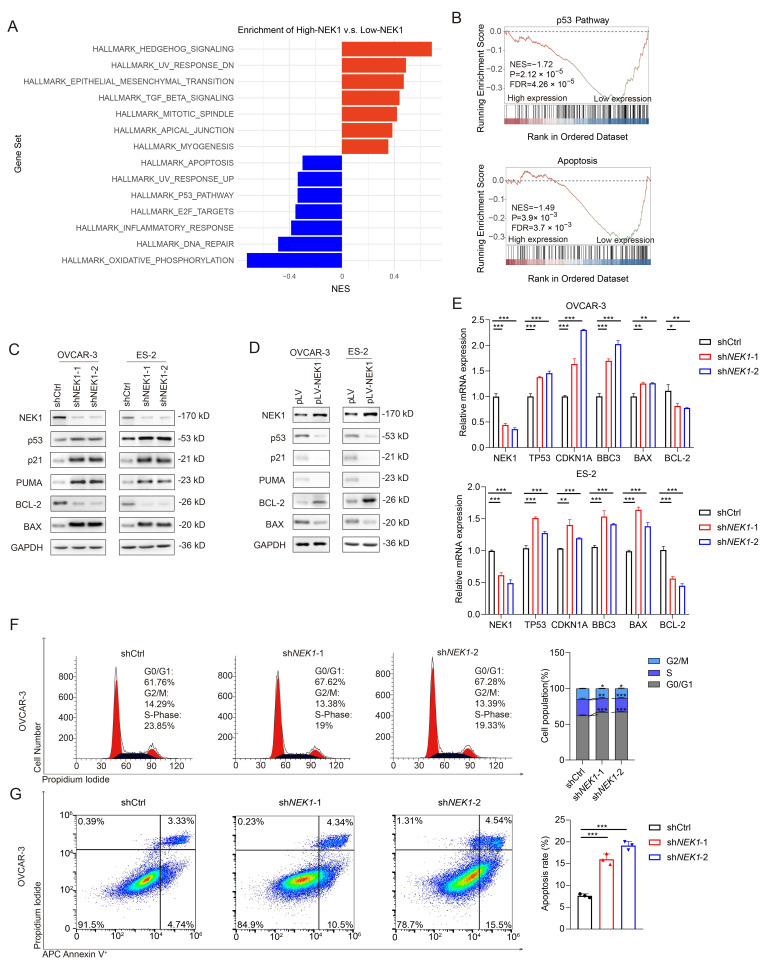
NEK1 attenuates p53-mediated cell cycle arrest and apoptosis in ovarian cancer. (**A**) Bar plot displaying selected Hallmark pathways identified by GSEA comparing high-NEK1 (*n* = 154) versus low-NEK1 (*n* = 154) TCGA-OV patients. The TCGA-OV cohort excluded samples with incomplete clinical annotations or missing survival data; 308 ovarian carcinoma samples with comprehensive clinical information were retained for GSEA analyses. The top 7 positively enriched pathways (activated in the high-NEK1 group) and selected negatively enriched pathways of biological interest (activated in the low-NEK1 group) are shown. The *x*-axis represents NES, with red bars indicating positive enrichment and blue bars indicating negative enrichment. The *y*-axis lists the Hallmark pathway names. (**B**) GSEA plots show the signaling pathways significantly enriched in the NEK1-high or NEK1-low group. (**C**) Effects of NEK1 knockdown on p53 and downstream cascades in OVCAR-3 and ES-2 cells determined by Western blotting assays. (**D**) Effects of NEK1 overexpression on p53 and downstream cascades in OVCAR-3 and ES-2 cells assessed by Western blotting assays. (**E**) qRT-PCR analysis of *TP53*, *CDKN1A* (p21), *BBC3* (PUMA), and *BAX* mRNA levels in OVCAR-3 and ES-2 cells with stable NEK1 knockdown and respective controls. Data are shown as mean ± standard deviation (SD) of three independent experiments. Two-tailed Student’s *t* test: * *p* < 0.05, ** *p* < 0.01, *** *p* < 0.001 vs. control. (**F**,**G**) Effects of NEK1 knockdown on the cell cycle transition (**F**) and apoptosis (**G**) determined by flow cytometry assays in OVCAR-3 cells.

The significant oncogenic role of NEK1 in OV cells prompted us to further investigate its underlying molecular mechanisms. We first performed Gene Set Enrichment Analysis (GSEA) using data from the TCGA-OV cohort, as described in Section 2.4. Subsequently, we conducted Western blotting and qRT-PCR to assess the activity of the p53 signaling pathway, following the methods outlined in Section 2.5 and Section 2.6. Furthermore, we utilized flow cytometry to analyze the cell cycle and apoptosis, adhering to the procedural steps detailed in Section 2.7 and Section 2.8. First, based on mRNA expression profile data of tumor samples from the TCGA-OV cohort (*n* = 308), we stratified them into high-NEK1 (*n* = 154) and low-NEK1 (*n* = 154) groups according to the median expression level. GSEA of the Hallmark gene collection revealed profound differences in pathway activities between these two groups. Although the oxidative phosphorylation pathway was ranked as the most significantly enriched negatively regulated pathway, the low-NEK1 group showed significant and widespread enrichment of DNA repair pathways and p53 signaling pathway simultaneously (Figure 4A,B). Activation of the p53 pathway is a key mechanism for restricting tumor growth, wherein p53 functions as a crucial tumor suppressor by regulating the cell cycle and inducing apoptosis [14,15]. These results suggest that NEK1 may promote the OV progression by reducing the activity of the p53 signaling pathway.

The tumor suppressor p53 governs cell cycle dynamics via p21-mediated cyclin-dependent kinase inhibition, suppressing the growth of genetically altered cells [27,28]. On the other hand, p53 can directly induce apoptosis by upregulating pro-apoptotic genes such as BAX and PUMA, or by suppressing anti-apoptotic genes such as BCL-2 [29,30,31]. To verify whether NEK1 influences the p53 pathway activity in OV cells, we evaluated the effects of NEK1 on the expression of p53 and its downstream effectors (p21, PUMA and BAX) by using Western blotting assays in OVCAR-3 and ES-2 cells. NEK1 knockdown markedly upregulated p53, p21, BAX, and PUMA expression. (Figure 4C). In contrast, anti-apoptotic BCL-2 levels exhibited substantial reduction (Figure 4C). Consistent with these findings, overexpression of NEK1 in OVCAR-3 and ES-2 cells notably suppressed p53 and its downstream signaling cascades, while significantly elevating the BCL-2 expression (Figure 4D). To determine whether the observed protein-level changes were accompanied by corresponding transcriptional alterations, we performed qRT-PCR analysis of p53 and its downstream target genes in NEK1-modulated OVCAR-3 and ES-2 cells. Consistent with the Western blotting results (Figure 4C,D), *NEK1* knockdown significantly upregulated *TP53* mRNA as well as the mRNA levels of *CDKN1A* (p21), *BBC3* (PUMA), and *BAX*, whereas *NEK1* overexpression produced the opposite effects (Figure 4E and Supplementary Figure S2A,B). These findings indicate that *NEK1* modulates the p53 signaling output through coordinated regulation of p53 transcript and protein abundance in ovarian cancer cells.

Subsequently, we further evaluated the effects of NEK1 on the cell cycle and apoptosis by flow cytometry assays. The results showed that knockdown of NEK1 significantly increased the G1 phase proportions in both OVCAR-3 and ES-2 cells, while reducing the S and G2/M phase proportions (Figure 4F, Supplementary Figure S3A). Additionally, NEK1 silencing markedly induced apoptosis in these OV cells (Figure 4G, Supplementary Figure S3B). The opposite phenomenon was observed upon NEK1 overexpression, manifesting as G1 phase depletion coupled with accumulation of cells in S and G2/M compartments. (Supplementary Figure S3C,D). Apoptosis rate also decreased significantly (Supplementary Figure S3E,F).

These findings collectively suggest that NEK1 substantially inhibits the activity of the p53 signaling pathway in OV cells, which may serve as a key molecular mechanism underlying NEK1-mediated promotion of tumor malignancy through attenuating cell cycle arrest and apoptosis.

### 3.5. NEK1 Exerts Its Oncogenic Role Depending on p53

**Figure 5:**
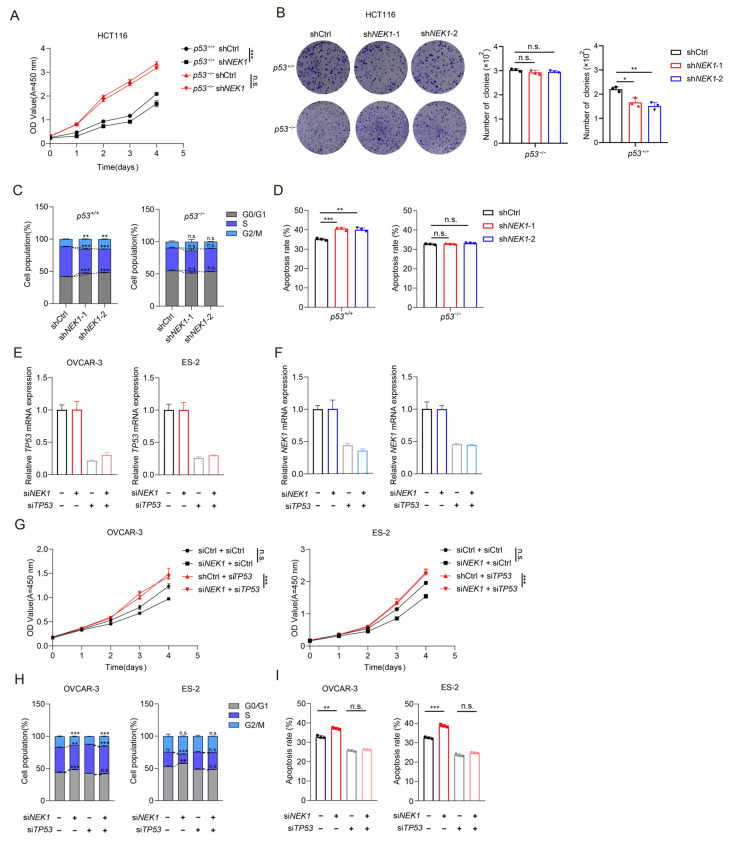
NEK1-driven oncogenicity is p53-context-dependent. (**A**,**B**) Using *p53^+/+^* (which contains functional p53) and *p53^−/−^* (p53-defective) HCT116 cells to generate stable cell lines with NEK1 knockdown and overexpression, as well as corresponding control groups (stable transfection with empty vector). (**A**) CCK-8 assay performed daily for 5 days. Data are mean ± SD of three independent experiments. Two-way ANOVA: *** *p* < 0.001 vs. shCtrl within each genotype; n.s., not significant between genotypes. (**B**) Colony-formation assays. The histogram shows mean ± SD colony number per well (*n* = 3). Unpaired Student’s *t* test: * *p* < 0.05, ** *p* < 0.01. (**C**,**D**) Knockdown of NEK1 increases the G1 arrest (**C**) and H_2_O_2_-induced apoptosis (**D**) in HCT116 *p53^+/+^* cells, but not in HCT116 *p53^−/−^* cells. Data are shown as mean ± standard deviation (SD) of three independent experiments, two-tailed Student’s *t* test. ** *p* < 0.01 vs. control, *** *p* < 0.001 vs. control. (**E**,**F**) qRT-PCR validation of siRNA-mediated knockdown efficiency in OVCAR-3 and ES-2 cells. (**E**) *TP53* mRNA levels following transfection with si*NEK1* or siCtrl. (**F**) NEK1 mRNA levels following transfection with si*TP53* or siCtrl. Data are mean ± SD of three independent experiments. (**G**) CCK-8 assay in OVCAR-3 and ES-2 cells co-transfected with si*NEK1*/si*TP53*. Data are mean ± SD of three independent experiments. Two-way ANOVA: *** *p* < 0.001 vs. siCtrl + siCtrl; n.s., not significant between si*NEK1* + si*TP53* and siCtrl + si*TP53*. (**H**) Quantification of cell cycle distribution in OVCAR-3 and ES-2 cells following co-transfection. G0/G1 phase proportions are shown as mean ± SD. ** *p* < 0.01, *** *p* < 0.001 vs. siCtrl + siCtrl; n.s., not significant between si*NEK1* + si*TP53* and siCtrl + si*TP53*. (**I**) Quantification of H_2_O_2_-induced apoptosis in OVCAR-3 and ES-2 cells following co-transfection. Apoptotic rates are shown as mean ± SD. ** *p* < 0.01, *** *p* < 0.001 vs. siCtrl + siCtrl; n.s., not significant between si*NEK1* + si*TP53* and siCtrl + si*TP53*. Representative flow cytometry profiles are provided in Supplementary Figure S4.

To confirm whether the oncogenic role of NEK1 is dependent on p53, we performed complementary experiments using *p53*^+/+^ (functional p53) and *p53*^−/−^ (p53-defective) HCT116 cells. As expected, CCK-8 assay results showed that NEK1 knockdown significantly inhibited cell growth in *p53*^+/+^ HCT116 cells (Figure 5A). In contrast, in *p53*^−/−^ HCT116 cells, the effects of NEK1 knockdown on cell growth were significantly attenuated, with no statistical difference observed between the two groups (Figure 5A). The results of clone formation assays were consistent with this observation that p53 defect significantly weakened the inhibitory effect of NEK1 knockdown on clone formation ability, and also blocked the promotion effect of NEK1 overexpression on colony formation ability (Figure 5B). Furthermore, flow cytometry analyses showed that NEK1 knockdown increased G0/G1 phase proportion and decreased S and G2/M phases in *p53*^+/+^ HCT116 cells, indicating cell cycle arrest in G1 phase. This effect was significantly blunted in *p53*^−/−^ HCT116 cells (Figure 5C). In contrast, NEK1 overexpression reduced G0/G1 arrest and promoted cell cycle progression in *p53*^+/+^ HCT116 cells, which was also reversed in *p53*^−/−^ HCT116 cells (Figure 5C). In terms of apoptosis induction (Figure 5D), the results showed a similar pattern: in *p53*^+/+^ HCT116 cells, NEK1 knockdown significantly enhanced H_2_O_2_-induced apoptosis; however, in *p53*^−/−^ HCT116 cells, there was no significant difference in apoptosis rate regardless of NEK1 expression level. These findings collectively confirm that the oncogenic functions of NEK1 in regulating cell cycle progression and apoptosis are dependent on the presence of p53.

To further validate the p53-dependency of NEK1-driven oncogenicity in an ovarian cancer context, we repeated the experiments in OVCAR-3 and ES-2 cells. Cells were co-transfected with si*NEK1* and either si*TP53* or a non-targeting control siRNA (siCtrl), and the effects on cell growth, cell cycle distribution, and apoptosis were systematically evaluated. qRT-PCR assays confirmed efficient knockdown of both *TP53* and *NEK1* transcripts (Figure 5E,F). In both OVCAR-3 and ES-2 cells, si*NEK1* alone significantly suppressed cell growth as measured by CCK-8 assays; however, knockdown of *TP53* abrogated this growth inhibition, with no significant difference between groups regardless of *NEK1* expression levels (Figure 5G). Flow cytometric analyses of cell cycle distribution revealed that si*NEK1*-induced G0/G1 arrest in OVCAR-3 and ES-2 cells was markedly reversed upon simultaneous *TP53* depletion, and no significant difference was observed compared to the *TP53*-knockdown-only group (Figure 5H). Regarding apoptosis, H_2_O_2_-induced cell death was significantly enhanced by si*NEK1* transfection in both ovarian cancer cell lines, whereas following *TP53* knockdown, apoptotic rates showed no significant difference regardless of *NEK1* status (Figure 5I). Collectively, these findings in OVCAR-3 and ES-2 cells recapitulate the p53-dependent oncogenic functions of NEK1 observed in HCT116 isogenic cells, thereby substantiating that NEK1 exerts its oncogenic effects through repressing p53 in an ovarian cancer cellular context.

### 3.6. NEK1 Sensitizes OV Cells to Genotoxic Treatment

Extensive studies have demonstrated that the p53 pathway plays a pivotal role in DNA-damage repair and cell-fate decisions [32,33]. Based on our prior findings that NEK1 negatively regulates p53, we hypothesized that NEK1 may modulate the sensitivity of OV cells to genotoxic treatment.

**Figure 6:**
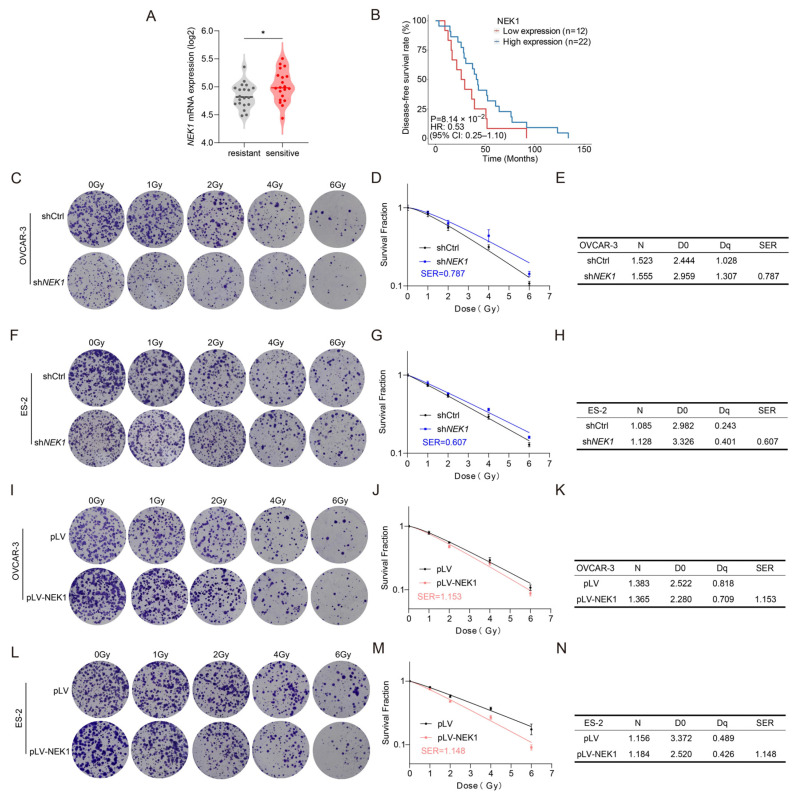
NEK1 increases the sensitivity of ovarian cancer cells to genotoxic treatments. (**A**) NEK1 mRNA levels in post-treatment biopsies of ovarian cancer patients who were subsequently treated with taxane/platinum. Patients were classified as sensitive (complete or partial response, *n* = 20) or resistant (stable or progressive disease, *n* = 20). Data were obtained from GSE63885. Violin plots show median ± interquartile range; * *p* < 0.05 vs. sensitive (Mann–Whitney U test). (**B**) Kaplan–Meier survival plots of OV patients treated with platinum/cyclophosphamide grouped by the expression of NEK1. The gene expression data and clinical information were obtained from the GSE63885 datasets. The HR and 95% CI were determined by Cox regression analysis, and the *p* value was calculated by the log-rank test. (**C**) Representative images of surviving colonies of NEK1-knockdown OVCAR-3 cells following irradiation (*n* = 3). (**D**) Survival curves for irradiated OVCAR-3 cells after NEK1 knockdown. (**E**) The multi-target single-hit model analysis of the values of N (Number of targets), D0 (Mean lethal dose), Dq (Quasi-threshold dose) and SER (Sensitivity Enhancement Ratio) in OVCAR-3 cells with NEK1 knockdown. (**F**) Representative images of surviving colonies of NEK1-knockdown ES-2 cells following irradiation (*n* = 3). (**G**) Survival curves for irradiated ES-2 cells after NEK1 knockdown. (**H**) The multi-target single-hit model analysis of the values of N, D0, Dq and SER in ES-2 cells with NEK1 knockdown. (**I**) Representative images of surviving colonies of NEK1 overexpression OVCAR-3 cells following irradiation (*n* = 3). (**J**) Survival curves for irradiated OVCAR-3 cells after NEK1 overexpression. (**K**) The multi-target single-hit model analysis of the values of N, D0, Dq and SER in OVCAR-3 cells with NEK1 overexpression. (**L**) Representative images of surviving colonies of NEK1 overexpression ES-2 cells following irradiation (*n* = 3). (**M**) Survival curves for irradiated ES-2 cells after NEK1 overexpression. (**N**) The multi-target single-hit model analysis of the values of N, D0, Dq and SER in ES-2 cells with NEK1 overexpression.

To this end, we first analyzed the transcriptomic datasets from the OV patients who received chemotherapy (GSE63885) [24,25]. We stratified patients treated with taxane and platinum chemotherapy strategy into chemotherapy-sensitive and chemotherapy-resistant groups according to their survival time. Notably, high NEK1 expression was significantly enriched in the favorable-responder cohort (*p* = 0.0368) (Figure 6A). Survival analysis of OV patients who received chemotherapy revealed that, compared to patients with low NEK1 expression, those with high NEK1 expression showed a trend toward improved overall survival (HR [95% CI] = 0.62 [0.36–1.06], *p* = 0.0814) (Figure 6B).

Furthermore, we performed functional experiments to confirm this observation in OV cells. OV cell lines were irradiated in vitro with ^60^Coγ-rays at a dose rate of 69 cGy/min, using radiation doses of 1, 2, 4, and 6 Gy. Colony formation assays showed that NEK1 knockdown significantly reduced radiosensitivity in OVCAR-3 and ES-2 cells, with sensitization-enhancement ratios (SERs) of 0.787 and 0.607, respectively (Figure 6C–H). In contrast, NEK1 overexpression produced a pronounced reduction in the number of surviving colonies and radioresistance of OVCAR-3 and ES-2 cells, with SERs of 1.153 and 1.148, respectively (Figure 6I–N). Consistent with the inhibitory role of NEK1 in the p53 signaling pathway, overexpression of NEK1 in OV cells increased the sensitivity to radiation by impairing DNA damage repair ability. Conversely, knockdown of NEK1 restored the function of the p53 pathway and enhanced cell viability, making cells more resistant to radiation. Consistent with our findings, previous studies have demonstrated that p53 expression levels are inversely correlated with susceptibility to genotoxic therapy [34,35]. To directly assess whether NEK1 affects DNA double-strand break (DSB) dynamics, we examined γH2AX levels by Western blot at early time points following ionizing radiation (IR, 6 Gy). In both OVCAR-3 and ES-2 cells, γH2AX signal peaked within 1 h post-IR and subsequently declined, consistent with active DSB repair. Notably, NEK1 knockdown significantly attenuated the γH2AX peak intensity and delayed its clearance kinetics, whereas NEK1 overexpression produced the opposite effect—enhancing γH2AX accumulation and accelerating its resolution (Supplementary Figure S5A,B). These opposing patterns suggest that NEK1 modulates DSB repair efficiency in a manner that depends on its expression level: high NEK1 promotes rapid DSB signaling and turnover, whereas NEK1 depletion impairs the initial damage response. Together, these findings demonstrate that NEK1-overexpressing ovarian cancers, characterized by attenuated p53 activity, display heightened radiosensitivity.

## 4. Discussion

The current standard of care for OV consists of cytoreductive surgery combined with platinum-based chemotherapy; however, relapse and chemoresistance limit the 5-year survival rate to approximately 47% [36]. Although the addition of bevacizumab to platinum/paclitaxel has been approved as first-line therapy, randomized trials have not demonstrated a significant improvement in overall survival [36]. Consequently, novel therapeutic strategies and molecularly targeted agents are urgently needed to increase cure rates. In the present study, we demonstrate that NEK1 accelerates OV progression by suppressing p53 and its downstream effectors, the cyclin-dependent kinase inhibitor p21 and the pro-apoptotic proteins BAX and PUMA. Flow-cytometric analyses revealed that NEK1 knockdown induces a pronounced G1 arrest and a marked increase in apoptosis. These findings provide experimental evidence that NEK1 represents a therapeutically tractable target in OV and provide a theoretical framework for future clinical development.

p53 governs cell cycle checkpoints, apoptosis and DNA damage repair, yet it is frequently inactivated in OV. Genomic mutation [37,38], excessive post-translational modification (e.g., acetylation or phosphorylation) [39] and aberrations in upstream regulators such as the E3 ubiquitin ligase MDM2 (Murine Double Minute 2) all contribute to p53 loss of function. Phosphorylation of MDM2 at threonines 166 and 186 enhances its nuclear localization, promotes p53 ubiquitination and thereby blunts p53-mediated apoptosis [40,41]. As a serine/threonine kinase, NEK1 is highly expressed in OV and may potentiate MDM2 phosphorylation, facilitating p53 degradation. While our data support a model in which NEK1 suppresses p53 signaling activity, the direct targets of NEK1 kinase activity (p53 versus MDM2) and the specific phosphorylation or ubiquitination events involved remain to be fully defined. Our qRT-PCR analysis (Figure 4E) revealed concordant changes in p53 mRNA and protein levels, indicating that NEK1 regulates p53 overall abundance through multilayered mechanisms that may involve transcriptional or post-transcriptional control, potentially in addition to effects on protein stability. Future studies employing in vitro kinase assays, MG132 proteasome inhibition, quantitative ubiquitination analysis, and proximity ligation assays will be necessary to establish the precise biochemical mechanism and determine whether NEK1 directly phosphorylates p53 or modulates MDM2 activity.

**Figure 7:**
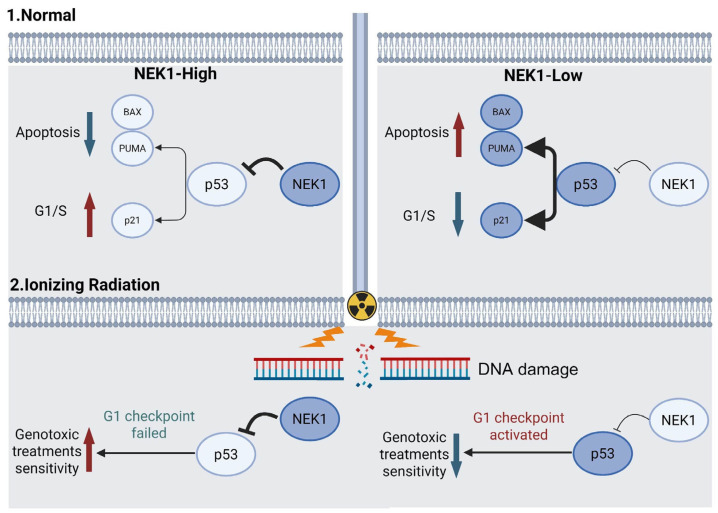
Proposed model illustrating the NEK1-p53 axis in OV progression and genotoxic treatment sensitivity. Normal conditions: In NEK1-high tumors (**left**), active NEK1 suppresses p53 protein levels, thereby downregulating p53 targets p21, BAX and PUMA while upregulating BCL-2. This attenuates G1 arrest and apoptosis, accelerates cell cycle progression, resulting in enhanced tumor growth. In NEK1-low settings (**right**), relief of p53 inhibition restores expression of p21 and BAX/PUMA, while diminishing BCL-2, leading to increased apoptosis and reduced tumor growth. (2) IR exposure: In NEK1-high cells (**left**), impaired p53 activity prevents G1 checkpoint activation. Cells with unrepaired DNA double-strand breaks (DSBs) progress into S phase, succumb to mitotic catastrophe, manifesting as genotoxic treatment sensitivity. In NEK1-low cells (**right**), p53 induces p21-mediated G1 arrest, allowing time for DNA repair, resulting in cell survival and genotoxic treatment resistance. Created in BioRender. huiyang, S. (2026) https://BioRender.com/a7tagah, accessed on 1 May 2026.

When combined with IR, our study uncovers a seemingly paradoxical NEK1-p53 regulatory axis within OV cells (Figure 7). Functionally, NEK1 suppresses p53 activity and attenuates its principal downstream arms, including cell cycle arrest (p21) and apoptosis initiation (PUMA/BAX). Consequently, under steady-state conditions, NEK1-high cells continuously enter S phase and evade apoptosis. This results in accelerated proliferation and enhanced tumorigenicity, correlating with poorer clinical prognosis. However, when exposed to IR, this pro-tumorigenic phenotype is inverted: NEK1-high cells, owing to impaired p53 activity, fail to activate p21-dependent G1 arrest and cannot eliminate irreparably damaged cells via the PUMA/BAX pathway. They therefore progress into mitosis with unrepaired DNA double-strand breaks and ultimately succumb to mitotic catastrophe. This was quantified in our clonogenic assays, where NEK1 overexpression significantly reduced colony survival, whereas NEK1 knockdown conferred radioresistance. Clinically, we found that high NEK1 expression was significantly enriched in chemotherapy-sensitive patients, supporting the predictive value of NEK1 for genotoxic treatment response. Although the clinical survival data did not reach statistical significance, the direction of effect is consistent with in vitro radiosensitivity findings, supporting the potential for further prospective validation of NEK1 as a predictive biomarker for genotoxic therapy.

PARP inhibitors (olaparib, niraparib, etc.) represent a cornerstone of OV treatment, particularly in *BRCA1/2*-mutated tumors, where they induce synthetic lethality by trapping PARP on DNA and preventing single-strand break repair [42,43,44]. However, p53 inactivation can rewire DNA-damage-repair pathways and potentially compromise PARP inhibitor efficacy. Our data caution against this approach in the context of concurrent genotoxic therapy: NEK1 knockdown restores p53-mediated DNA repair and enhances cell viability under radiation exposure, effectively converting sensitive tumors into resistant ones. Therefore, the combination of NEK1 inhibitors with PARP inhibitors or other DNA-damaging agents may be counterproductive in NEK1-high tumors.

Several limitations should be noted. The xenograft study employed a single cell line with NEK1 overexpression only, used a subcutaneous rather than orthotopic model, and lacked IHC validation of p53 pathway markers (p53, p21, TUNEL) due to time constraints. Future work will address these gaps through intraperitoneal models with both gain- and loss-of-function, comprehensive IHC, and detailed biochemical characterization of the NEK1-p53 interaction. In addition, an important limitation of this study is the insufficient statistical power of the clinical survival data. The chemotherapy survival analysis in the GSE63885 cohort (*n* = 40, event numbers not reported) had limited statistical power. While the *p* = 0.0814 result is directionally consistent with our in vitro findings of NEK1-enhanced radiosensitivity, it cannot serve as definitive confirmation for clinical efficacy prediction. Larger prospective cohorts or clinical trials are required to validate the clinical utility of NEK1 as a stratification biomarker for genotoxic therapy. Finally, while our data support a model where NEK1-mediated p53 suppression contributes to radiosensitivity, we acknowledge that NEK1 itself functions as a DNA damage response kinase that regulates ATR phosphorylation and non-homologous end joining (NHEJ). Therefore, the observed radiosensitization may involve both p53-dependent (checkpoint impairment) and p53-independent (direct DNA repair modulation) mechanisms.

Collectively, this study has direct clinical implications. NEK1 expression could serve as a predictive biomarker to stratify OV patients for genotoxic therapy. Tumors with high NEK1 levels may exhibit enhanced sensitivity to IR and platinum-based chemotherapy, whereas NEK1-low tumors may demonstrate relative resistance. Furthermore, our results suggest that therapeutic strategies modulating NEK1 activity warrant investigation. In NEK1-high tumors, standard genotoxic regimens may be sufficient, whereas in NEK1-low tumors, where p53 function is relatively intact, combination approaches targeting the p53 pathway or alternative DNA repair mechanisms may be required to overcome radioresistance.

## 5. Conclusions

In summary, this study demonstrates that NEK1 functions as an oncogenic driver in ovarian cancer by suppressing the p53 signaling pathway, leading to decreased expression of p21, BAX, and PUMA, which consequently attenuates cell cycle arrest and apoptosis while promoting proliferation, migration, invasion, and tumor growth. Clinically, elevated NEK1 expression serves as an independent prognostic biomarker associated with poor patient survival across multiple ovarian cancer cohorts. Notably, despite its pro-tumorigenic role under normal conditions, high NEK1 expression paradoxically enhances sensitivity to ionizing radiation and chemotherapy by impairing p53-mediated DNA damage repair, revealing a context-dependent dual function. These findings establish NEK1 as a promising therapeutic target and suggest that NEK1 expression levels could guide patient stratification for genotoxic therapies. The dependence of NEK1 oncogenic activity on p53 further underscores the importance of considering p53 status in future therapeutic strategies, potentially enabling more personalized treatment approaches for ovarian cancer patients.

## Data Availability

All data supporting the findings of this study are publicly available and were downloaded from the following repositories: TCGA via the Genomic Data Commons (https://portal.gdc.cancer.gov). GEO under accession numbers GSE26712, GSE63885 and GSE165808 (https://www.ncbi.nlm.nih.gov/geo (accessed on 1 May 2026)).

## Supplementary Materials

The following supporting information can be downloaded at: https://www.mdpi.com/article/10.3390/cimb48050486/s1.

## Abbreviations

The following abbreviations are used in this manuscript:

OV	Ovarian cancer
NEK1	NIMA-related kinase 1
GSEA	Gene set enrichment analysis
CCA	Cervical cancer
CRC	Colorectal cancer
IR	Ionizing radiation
TCGA	The Cancer Genome Atlas
GEO	Gene Expression Omnibus
OS	Overall survival
SER	Sensitization-enhancement ratio
OD	Optional density
IHC	Immunohistochemistry
