# Supplementary material for: NEK1 Promotes Ovarian Cancer Progression via p53 Suppression While Enhancing Sensitivity to Genotoxic Therapy
Source: Curr Issues Mol Biol. 2026 May 7;48(5):486. doi: 10.3390/cimb48050486 (PMC13206693; doi:10.3390/cimb48050486)
Supplement: Supplementary file 1 [file cimb-48-00486-s001.zip › Supplemental materials .pdf]

Supplemental Materials

Index

1. Supplemental Figures

Supplemental Figure S1. NEK1 increases the migration and invasion abilities of OV cells.

Supplemental Figure S2. NEK1 overexpression suppresses p53 signaling and downstream targets at the transcriptional levels.

Supplemental Figure S3. NEK1 regulates cell cycle progression and apoptosis in OV cells.

Supplemental Figure S4. Representative flow cytometry profiles of cell cycle and apoptosis analyses in HCT116, OVCAR-3 and ES-2 cells.

Supplemental Figure S5. NEK1 modulates γH2AX dynamics following ionizing radiation.

2. Supplemental Tables

Supplemental Table S1. The shRNAs, siRNAs and primers used in this study.

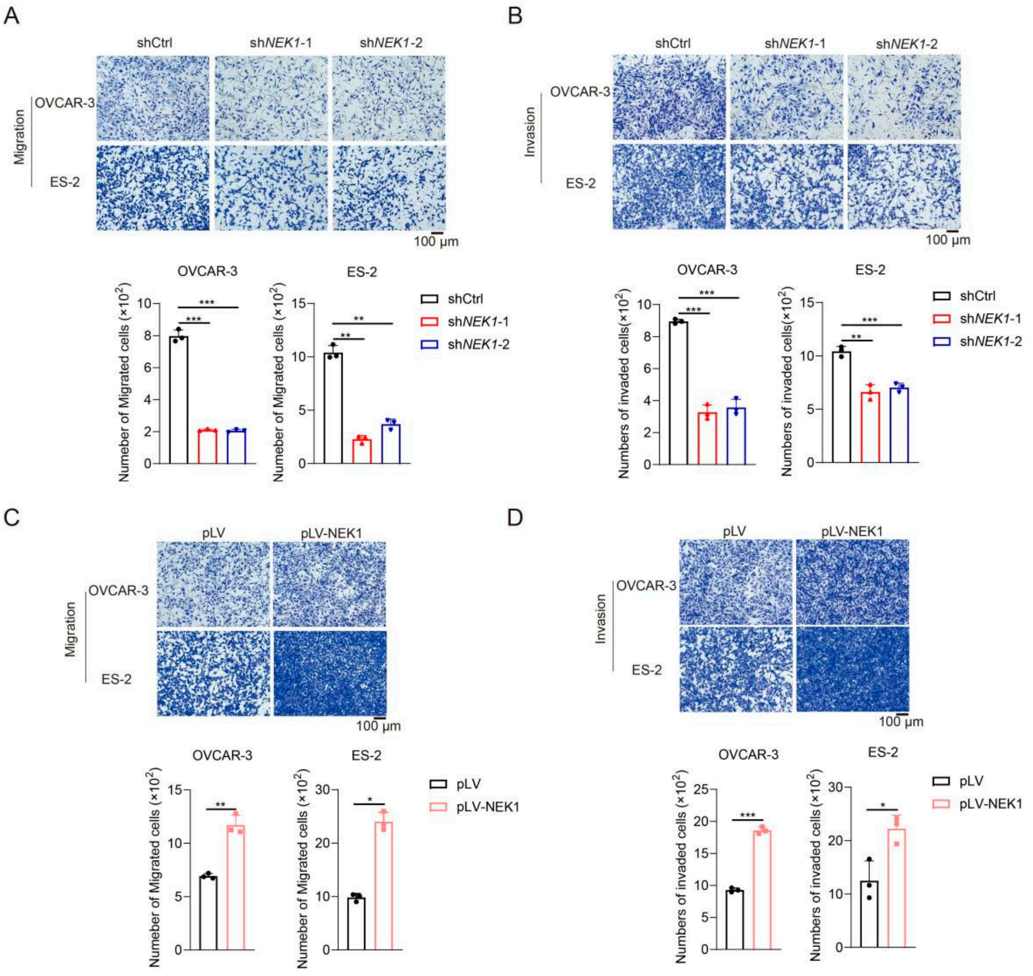

Supplemental Figure S1. NEK1 increases the migration and invasion abilities of OV cells.

(**A**) Effects of NEK1 knockdown on the migration of OVCAR-3 and ES-2 cells.

(**B**) Effects of NEK1 knockdown on the invasion of OVCAR-3 and ES-2 cells.

(**C**) Effects of NEK1 overexpression on the migration of OVCAR-3 and ES-2 cells.

(**D**) Effects of NEK1 overexpression on the invasion of OVCAR-3 and ES-2 cells.

Representative images of migration and invasion assays are shown in the upper section. Scale bar, 100 μm. Data are shown as mean ± standard deviation (SD) of three independent experiments, two-tailed Student's *t* test. **P* < 0.05 vs. control, ***P* < 0.01 vs. control, ****P* < 0.001 vs. control.

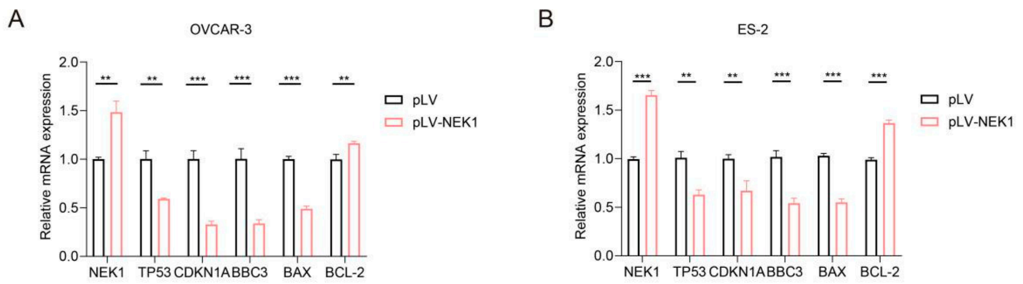

Supplemental Figure S2. NEK1 overexpression suppresses p53 signaling and downstream targets at the transcriptional levels.

(**A-B**) qRT-PCR analysis of TP53, CDKN1A (p21), BBC3 (PUMA), and BAX mRNA levels in OVCAR-3 (**A**) and ES-2 (**B**) cells with stable NEK1 overexpression and respective controls. Data are shown as mean ± standard deviation (SD) of three independent experiments. Two-tailed Student's t test: *$P < 0.05$, **$P < 0.01$, ***$P < 0.001$ vs. control.

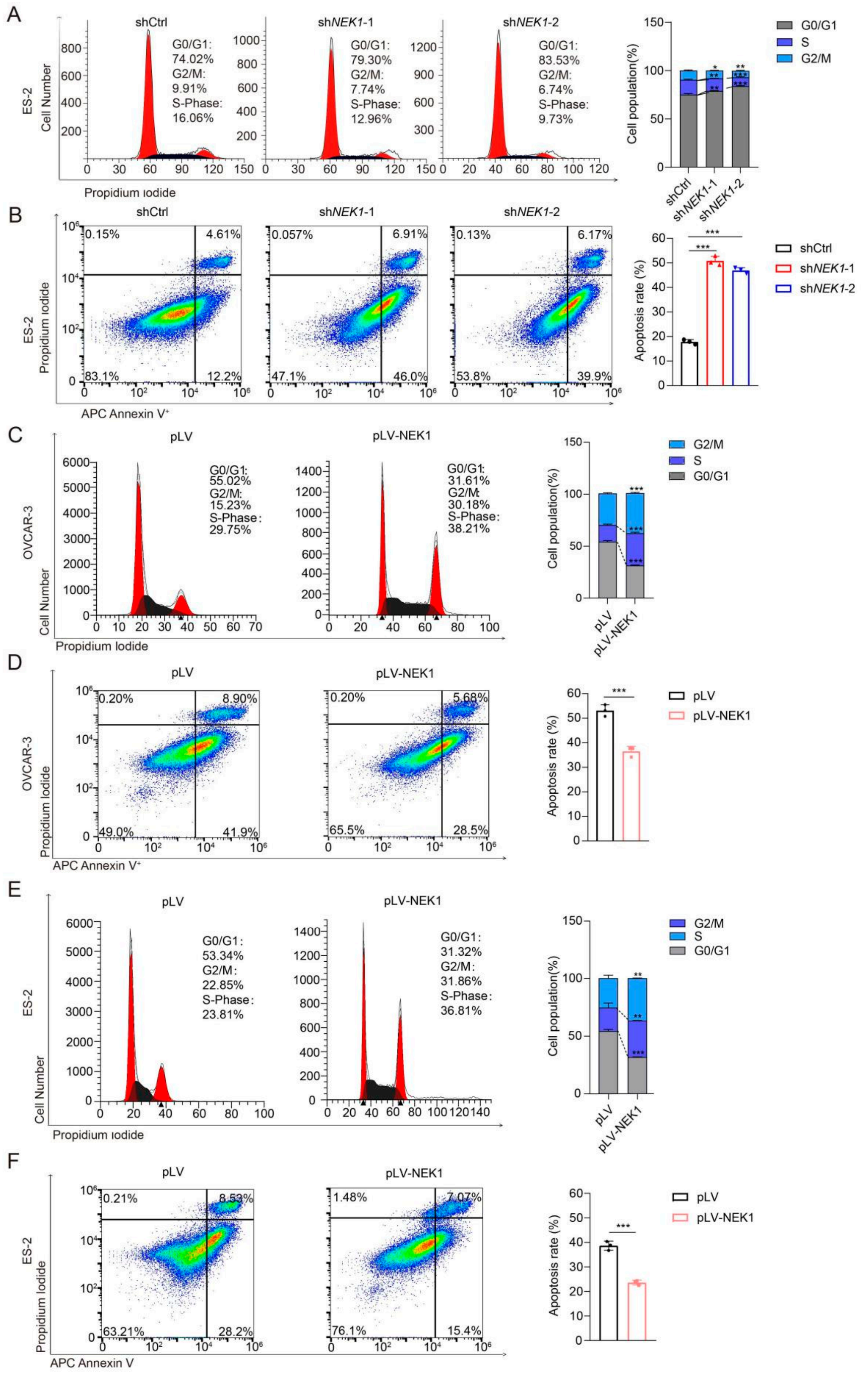

Supplemental Figure S3. NEK1 regulates cell cycle progression and apoptosis in OV cells.

(**A**) Effects of NEK1 knockdown on the cell cycle transition determined by flow

cytometry assays in ES-2 cells.

(**B**) Effects of NEK1 knockdown on the cell apoptosis determined by flow cytometry assays in ES-2 cells.

(**C**) Effects of NEK1 overexpression on the cell cycle transition determined by flow cytometry assays in OVCAR-3 cells.

(**D**) Effects of NEK1 overexpression on the cell apoptosis determined by flow cytometry assays in OVCAR-3 cells.

(**E**) Effects of NEK1 overexpression on the cell cycle transition determined by flow cytometry assays in ES-2 cells.

(**F**) Effects of NEK1 overexpression on the cell apoptosis determined by flow cytometry assays in ES-2 cells. Data are shown as mean ± standard deviation (SD) of three independent experiments, two-tailed Student's t test. *$P < 0.05$ vs. control, **$P < 0.01$ vs. control, ***$P < 0.001$ vs. control.

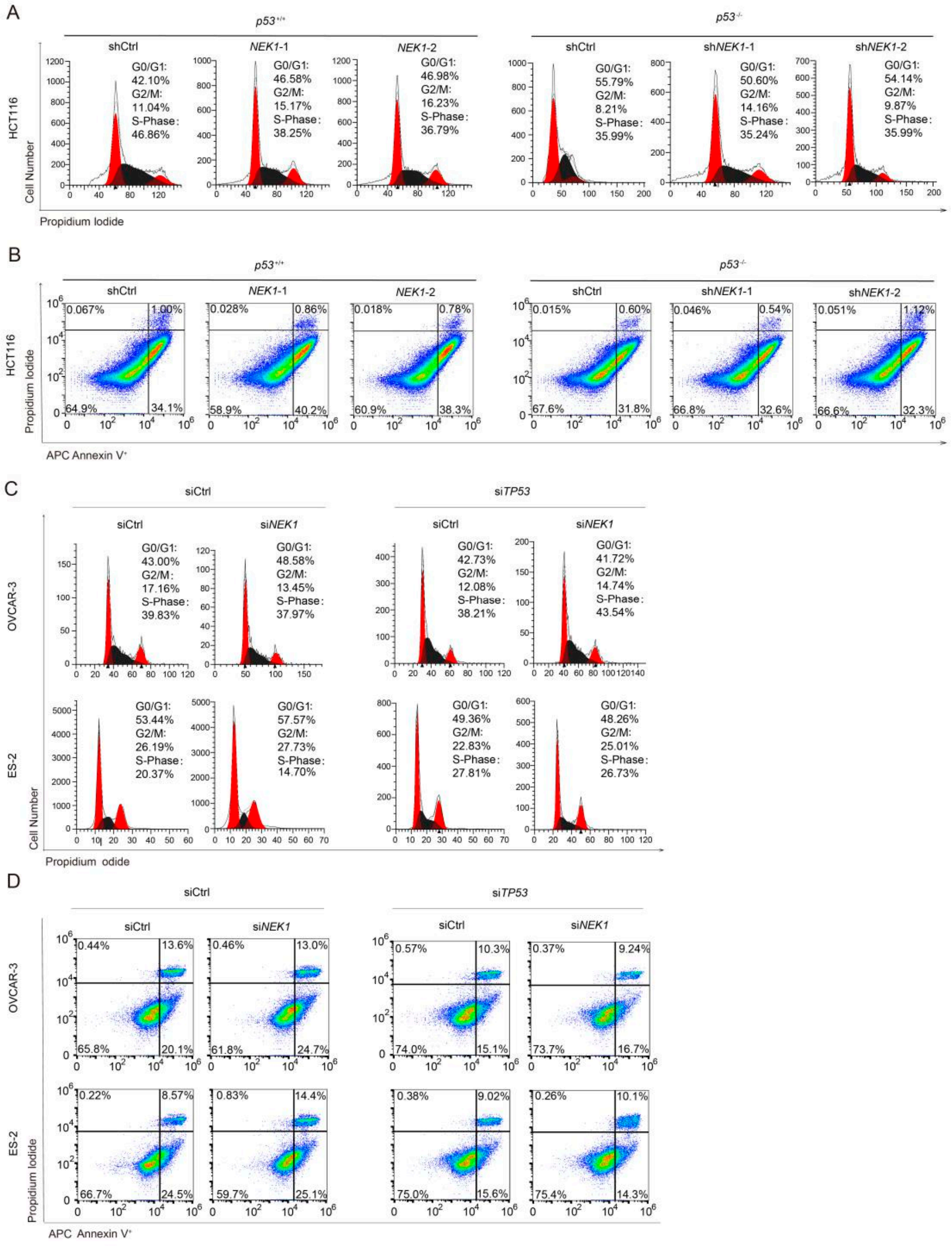

Supplemental Figure S4. Representative flow cytometry profiles of cell cycle and apoptosis analyses in HCT116, OVCAR-3 and ES-2 cells.

(A) Cell cycle distribution profiles of HCT116 *p53*$^{+/+}$ and *p53*$^{-/-}$ cells with stable NEK1 knockdown or overexpression, stained with propidium iodide and analyzed by flow cytometry.

(B) Apoptosis profiles of HCT116 *p53*$^{+/+}$ and *p53*$^{-/-}$ cells with stable NEK1 knockdown or overexpression, following H_2O_2 treatment and dual staining with Annexin V-APC and propidium iodide.

(C) Cell cycle distribution profiles of OVCAR-3 and ES-2 cells co-transfected with si*NEK1* + siCtrl, si*NEK1* + si*TP53*, siCtrl + si*TP53* or siCtrl + siCtrl, stained with propidium iodide and analyzed by flow cytometry.

(D) Apoptosis profiles of OVCAR-3 and ES-2 cells co-transfected as above, following H_2O_2 treatment and dual staining with Annexin V-APC and propidium iodide.

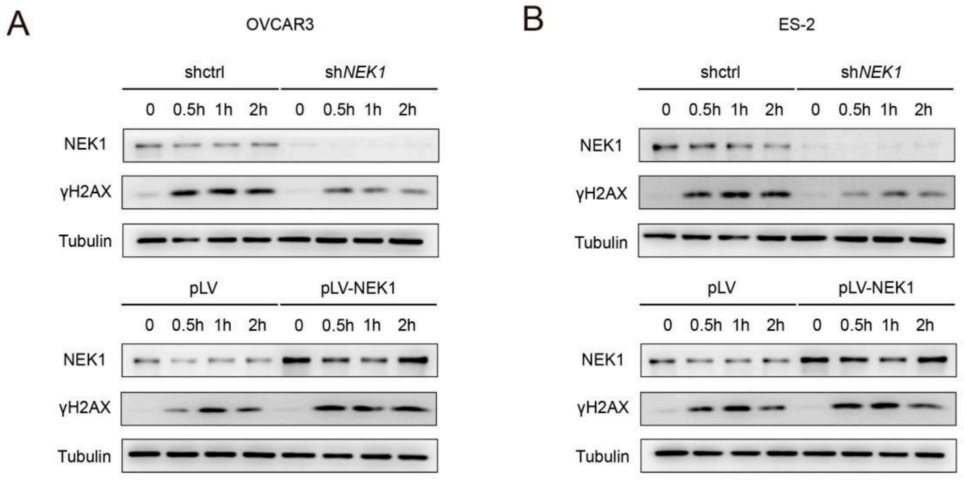

Supplemental Figure S5. NEK1 modulates γH2AX dynamics following ionizing radiation.

(**A–B**) Western blot analysis of γH2AX in OVCAR-3 (**A**) and ES-2 (**B**) cells at indicated time points after 6 Gy IR. Cells were stably transduced with shNEK1, NEK1-overexpression, or respective controls.

Supplemental Table S1. The siRNAs, shRNAs and primers used in this study.

Names	Sequences (5'→3')
Control shRNA	ATCGACTAGCCACTTAGAC
NEK1 shRNA-1	TGGAATACCTTTAGCATATAA
NEK1 shRNA-2	TACAAAGCCTGCCGCTAAATA
Negative control siRNA	UUCUCCGAACGUGUCACGUdTdT
NEK1 siRNA-1	GCAGCUAGCUGUAGAAAGAdTdT
NEK1 siRNA-2	GGACCAACUUAGUGACAUUdTdT
TP53 siRNA-1	GAAUGAGGCCUUAGAGUUAdTdT
TP53 siRNA-2	CCAUCUACAAGAAGUCACAdTdT
NEK1-F	TCCTTTTCTGGGCAGTGGAG
NEK1-R	TGCCCTTTTTGCCTTTCTGG
TP53-F	TGTGACTTGCACGTACTCCC
TP53-R	ACCATCGCTATCTGAGCAGC
CDKN1A-F	GCCGAAGTCAGTTCCTTGTG
CDKN1A-R	TTCTGACATGGCGCCTCCT
BBC3-F	GGGGAAAGTGAAAGAGGGAGG
BBC3-R	TCCCTGGGGCCTTCCAGT
BAX-F	GGGGAGCAGCCCAGAGG
BAX-R	CGATCCTGGATGAAACCCTGA
BCL-2-F	GGATAACGGAGGCTGGGATG
BCL-2-R	TGACTTCACTTGTGGCCCAG
GAPDH-F	GGGAGCCAAAAGGGTCATCA
GAPDH-R	TGATGGCATGGACTGTGGTC
